# Vegetarian Diet Is Associated with Lower Risk of Depression in Taiwan

**DOI:** 10.3390/nu13041059

**Published:** 2021-03-24

**Authors:** Yu-Chih Shen, Chiao-Erh Chang, Ming-Nan Lin, Chin-Lon Lin

**Affiliations:** 1 Department of Psychiatric, Hualien Tzu Chi Hospital, Buddhist Tzu Chi Medical Foundation, Hualien 970, Taiwan; shengmp@gmail.com; 2 Institute of Epidemiology and Preventive Medicine, College of Public Health, National Taiwan University, Taipei 100, Taiwan; d03849010@ntu.edu.tw; 3 Department of Family Medicine, Dalin Tzu Chi Hospital, Buddhist Tzu Chi Medical Foundation, Chiayi County 622, Taiwan; mingnan.lin@gmail.com; 4 Department of Family Medicine, College of Medicine, Tzu Chi University, Hualien 970, Taiwan; 5 Department of Cardiology, Dalin Tzu Chi Hospital, Buddhist Tzu Chi Medical Foundation, Chiayi County 622, Taiwan; 6 Department of Internal Medicine, College of Medicine, Tzu Chi University, Hualien 970, Taiwan

**Keywords:** vegetarian diet, plant-based diet, depression, taiwanese, buddhist

## Abstract

In order to determine whether Taiwanese vegetarian diets reduce the risks of depression, we analyzed data from the Tzu Chi Vegetarian Study (TCVS), which is a prospective cohort study following 12,062 participants from the Buddhist Tzu Chi Foundation of Taiwan since 2005. The cohort was prospectively followed by linking to the National Health Institute Research Database (NHIRD) of Taiwan and hazard ratios of depression between vegetarian and non-vegetarian groups were calculated by Cox proportional hazards regression. We assessed dietary intake using a detailed food frequency questionnaire (FFQ). Incident depression was ascertained through linkage to NHIRD which had claim records with the International Classification of Diseases, and a total of 3571 vegetarians and 7006 non-vegetarians were included in this analysis. Compared with non-vegetarians, the vegetarian group had a lower incidence of depressive disorders (2.37 vs. 3.21 per 10,000 person-years; adjusted hazard ratio (aHR): 0.70; 95% confidence interval (95% CI): 0.52–0.93). Thus, Taiwanese vegetarians had a lower risk of developing subsequent depressive disorders compared with non-vegetarians. This indicated that diet may be an important measure for the prevention of depression. However, to generalize to the global population requires further study.

## 1. Introduction

Depression is a common mental disorder and one of the main causes of disability worldwide [1]. In the Global Burden of Disease Study of 2010, major depressive disorder (MDD) was the second leading cause of all Years Lived with Disability (YLDs) globally [2]. The prevalence of major depression varies widely across different countries and with cultural backgrounds [3]. The prevalence in Taiwan appeared low compared to most Western countries and is attributed to cultural stoicism with low help-seeking behavior [4].

MDD is associated with many chronic medical conditions, such as chronic pulmonary disease, asthma, migraine, arthritis, heart disease, hypertension, and back pain [5]. Depressive disorders carry significant morbidity and mortality with a reduction in life expectancy [6]. The high rates of medical comorbidities, in addition to a high risk of suicide, are thought to be reasons for the decrease in life expectancy seen in depressive disorders [6,7].

In the field of nutritional psychiatry, systematic reviews have shown that healthy dietary patterns such as Mediterranean diets (high intake of vegetables, fruit, wholegrains, nuts, seeds, and fish, with limited processed foods) are inversely associated with the risk of depression [8,9,10,11]. In contrast, diets high in processed, fat, sugar foods are associated with depression and anxiety [8,11,12].

Vegetarian diets lower the risk of certain chronic diseases, such as cardiovascular diseases, cardiometabolic risk factors, some cancers, and total mortality [8] raising questions about whether the potential benefits extend to depressive disorders. Indeed, in a meta-analysis, Lai et al. concluded that high intakes of fruit, vegetables, fish, and whole grains are associated with a reduced depression risk [12].

However, mood studies in vegetarians have yielded contradictory results, either demonstrating mood protection or increased risks of depression. In a study of teens in the United States, vegetarians were more likely to have considered or attempted suicide [13]. In another study of Australian women in their twenties, a higher percentage of vegetarians reported depression as compared to non-vegetarians [14]. In a study of adults in the German community, vegetarians showed a high prevalence of depressive disorders, but the adoption of the vegetarian diet followed the onset of depressive disorders [15]. In a study of adult men in England, self-identification as a vegetarian was associated with an increased risk of depressive symptoms [16]. All these studies are cross-sectional associations, and nutritional deficiencies (especially vitamin B12 and iron) have been mentioned as potential causes, but reverse causation cannot be excluded.

In a meta-analysis [17] (four cohort studies and nine cross-sectional studies) assessing the relationship between the consumption of a vegetarian diet and depression, anxiety, and stress, no association between the consumption of a vegetarian diet and depression was demonstrated from the pooled data.

In another meta-analysis [18], no statistically significant differences were found between vegetarians and omnivores regarding the incidence of depression. Although subgroup analysis showed a statistically significant higher depression level in vegetarians/vegans under 26 years old, heterogeneity among studies was very high. In another study of vegetarian diet and mental health in culturally diverse samples [19], vegetarianism was not associated with mental health in the US, Russia, or Germany, but was associated with anxiety and depression in Chinese students, who self-reported being vegetarian, at the rate of 22%, as compared with 2.8–16% in the other samples. Moreover, their family affluence score was lower than other samples, indicating that reasons for being vegetarian might differ from other samples due to ethnic, cultural, or economic factors.

On the other hand, in a cross-sectional study of Seventh-day Adventist adults in the United States (members of a Christian denomination), vegetarian Adventists reported fewer negative emotions than non-vegetarian Adventists [20]. Furthermore, a pilot randomized controlled trial demonstrated that restricting meat, fish, and poultry for two weeks significantly improves mood state among omnivores [21]. In a 24-week trial study of diabetic patients, mood (assessed using the Beck Depression Inventory) improved much more for participants in the vegetarian diet group compared to those following the standard diabetic diet [22].

Previous studies mostly depend on self-reported questionnaires. No studies to date, to our knowledge, have used clinical diagnoses of depressive disorders by physicians based on medical care data. In addition, prospective studies tend to be short-term [21,22], and long-term follow-up comparison is not available. The present study aims to determine prospectively whether a vegetarian diet increases or decreases the risk of depressive disorders by using the claims data from the National Health Insurance (NHI) program of a large cohort with a high percentage of vegetarians in Taiwan.

## 2. Materials and Methods

### 2.1. The Tzu Chi Vegetarian Study (TCVS)

The TCVS recruited 12,062 participants from the Buddhist Tzu Chi Foundation. These volunteers were devoted Buddhists who spent a substantial amount of time volunteering for Tzu Chi’s projects involving community charity work, local and international disaster relief, recycling and environmental conservation activities, and hospital volunteer work. These volunteers had undergone at least two years of training and were required to quit smoking or alcohol drinking habits before becoming certified as Tzu Chi volunteers. Volunteers were also encouraged to consume a vegetarian diet as frequently as possible, as a way to conserve the environment and practice compassion toward animals (according to Buddhist teachings).

In the year 2005, community volunteer leaders helped distribute the research questionnaires to their team volunteer members, collect, and mail back the questionnaires to the research team. The questionnaires included sections on basic demographics, medical history, lifestyle (smoking, alcohol drinking, and exercise habits), and diet. The study was approved by the Institutional Review Board of Dalin Tzu Chi hospital and all participants gave written informed consent.

### 2.2. Exclusion Criteria

**Figure 1:**
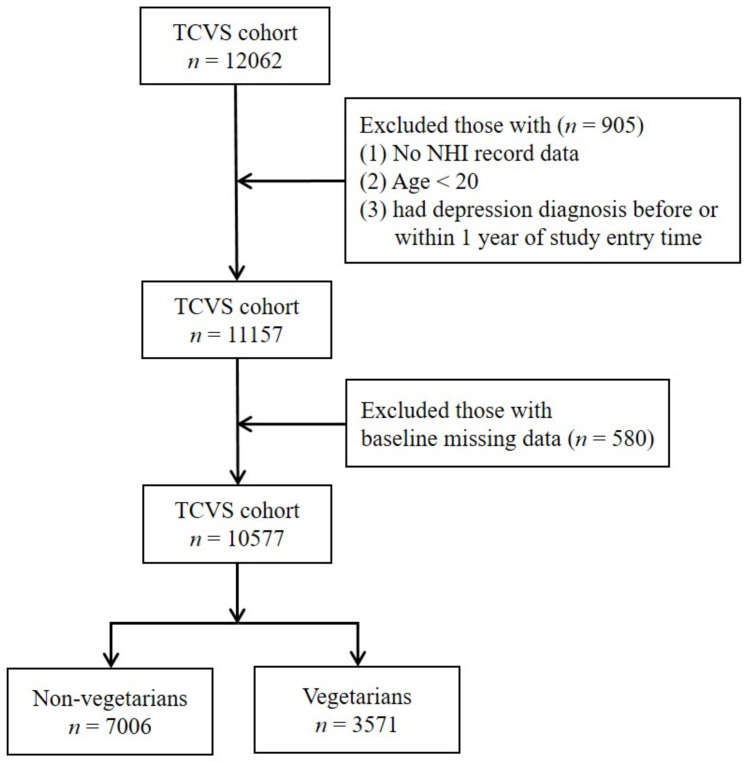
The flowchart of data processing.

Participants with age less than 20, who had depression diagnosis before or within one year of study entry time were excluded. We then further excluded those with missing data on covariates (sex and education levels). The detailed steps are outlined in Figure 1.

### 2.3. Case Ascertainment

Baseline data were linked to the National Health Institute Research Database (NHIRD) of Taiwan and the National Death Registry at the Health and Welfare Data Science Center (HWDSC), Ministry of Health of Taiwan, through a unique personal identification number. As a government regulation to protect the privacy of the participants’ personal data, all analyses were performed in the HWDSC, and only summarized results could be released. The NHI coverage of the Taiwanese population increased from 96% in the year 2001 to nearly 100% in the year 2010 [23,24]. Participants were followed-up until 31 December 2014. Incident cases of depression were identified if a participant had at least two outpatient or one inpatient admission to the psychiatric department, with International Classification of Diseases, 9th Revision, Clinical Modification (ICD-9-CM) codes of 296.2, 296.3, 300.4, and 311 as the diagnosis.

### 2.4. Assessment of Diet and Covariates

Diet was assessed through a 57-item food frequency questionnaire (FFQ) adopted from the one for the Nutrition and Health Survey in Taiwan (NAHSIT). A similar FFQ had later been validated among certified Tzu Chi volunteers and showed good reliability (through repeated measurements) and validity (with repeated dietary records and biomarkers) [25]. Participants were classified as vegetarians if they self-reported as vegetarians in a question asking vegetarian status, and reported “no eating” in frequency questions for all individual meat and fish items in the FFQ. Those who reported “eating” meat or fish in the FFQ were classified as non-vegetarians. Comorbidities including hypertension, diabetes mellitus, and hyperlipidemia were assessed through the NHI database based on ICD-9-CM codes, 401–405, 250, and 272, respectively.

### 2.5. Statistical Analysis

Independent sample t-test and Chi-square test were used to compare baseline characteristics between vegetarians and non-vegetarians. Follow-up time was computed from study entry to the date of diagnosis for depression, date of death from any cause, or end of the study period (31 December 2014), whichever came first. Cox proportional hazards regression was used to compare the incidence of depression between vegetarians and non-vegetarians, with adjustment for age, sex, educational level, marital status, exercise habit, tobacco, and alcohol consumption, and physical comorbidities (hypertension, diabetes mellitus, and hyperlipidemia). Proportional hazard assumption was tested through (1) visual inspection of survival curves (clear separation in the curves of vegetarians and non-vegetarians) and (2) test of interaction terms between each variable and time in the model. The results showed no sign of proportionality violation. All statistical analyses were performed using SAS version 9.4 (SAS Institute, Cary, NC, USA).

## 3. Results

Table 1 showed the baseline social–demographic characteristics of vegetarians and non-vegetarians. A total of 3571 vegetarians and 7006 non-vegetarians were included in this analysis. Compared with non-vegetarians, vegetarians were slightly older (51.5 vs. 50.4 year-old) with a higher proportion of females (74.0% vs. 61.9%). The distributions of education level and marital status were significantly different between the two groups. Compared with non-vegetarians, vegetarians were less likely to do regular exercise, smoke, or drink alcohol. In terms of comorbidities, vegetarians were less likely to have hypertension, diabetes mellitus, and hyperlipidemia. The mean follow-up years were 9 years.

**Figure 2:**
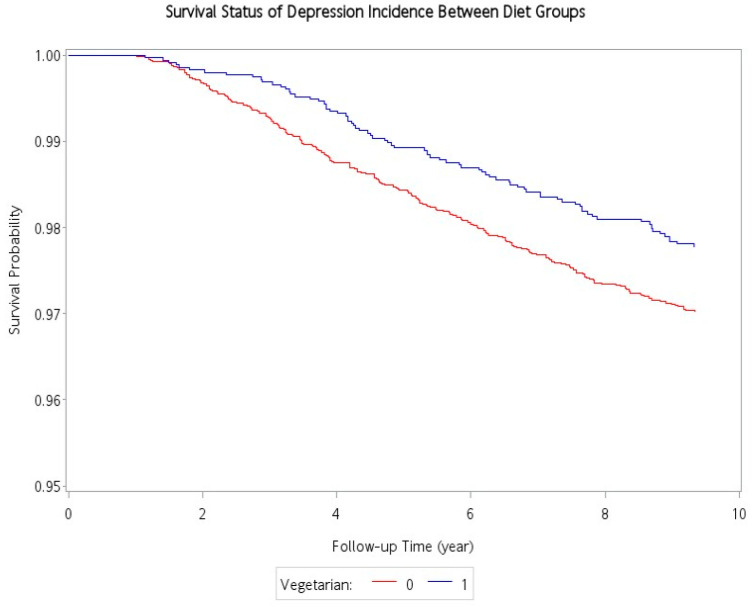
Cumulative incidence of depression for vegetarians and non-vegetarians.

Table 2 shows the association between incident depression and various potential risk factors. There was a total of 284 incident cases of depression occurred during the follow-up period (78 and 206 incident cases of depression in the vegetarians and non-vegetarians, respectively). Compared with non-vegetarians, the vegetarian had a protective association with incident depressive disorders (2.37 vs. 3.21 per 10,000 person-years). After adjustment for age, sex, education level, marital status, exercise habits, smoking, alcohol drinking, and comorbidities, the adjusted hazard ratio (aHR) was: 0.70; 95% confidence interval (95% CI): 0.52–0.93. A Kaplan–Meier plot demonstrated that the cumulative incidence curve of depressive disorders was lower in the vegetarian group than in the non-vegetarian group, and the log-rank test between the two survival curves was significant (*p* = 0.02) (Figure 2). Male sex was protective against depression (aHR: 0.58, 95% CI: 0.41–0.83). In contrast, lower education (below elementary school) (aHR: 1.59, 95% CI: 1.07–2.38), and having comorbidities including hypertension (aHR:1.40, 95% CI: 1.05–1.86), diabetes mellitus (aHR:1.49, CI: 1.10–2.00), and hyperlipidemia (aHR:1.34, 95% CI: 1.00–1.78) were significantly associated with an increased risk for incident depression in adjusted analyses.

Table 3 shows the stratification analysis. For the subgroups aged under 50 years, attained tertiary education or above, married, non-smokers, and non-drinkers, vegetarian diets were significantly protective against depressive disorder. On the other hand, although vegetarian diets were generally protective (adjusted hazard ratio less than 1) against depressive disorder in the other subgroups, they are not statistically significant, most likely as a result of small case numbers. Test for interaction showed no statistical significance as demonstrated by the *p*-value for the interaction of all subgroups.

## 4. Discussion

The main finding of this study is that in our cohort of Taiwanese Buddhists, vegetarians had a lower risk of developing subsequent depressive disorders compared to non-vegetarians (adjusted hazard ratio of 0.7). In addition, further stratification analysis revealed that the association between a vegetarian diet and incident depression was consistent across subgroups of age and sex as a protective trend.

Our study results are consistent with other published studies. Sanchez et al. reported in a large cohort study that better adherence to the Mediterranean diet was associated with a reduced risk of depression among Spanish adults [26]. Psaltopoulou et al. [10] reported in a meta-analysis that high adherence to the Mediterranean diet was consistently associated with reduced risk for depression in all types of studies (longitudinal cohort, case-control, and cross-sectional) both in the Mediterranean and non-Mediterranean countries (relative risk (RR): 0.68, 95% CI: 0.54–0.86), in addition to stroke (RR: 0.71, 95% CI: 0.57–0.89), and cognitive impairment (RR: 0.60, 95% CI: 0.43–0.83). Even the degree of protection (30%) is quite similar. Lassale et al. [27] also reported in a meta-analysis that adhering to a healthy diet, particularly a traditional Mediterranean diet, appears to confer protection against depression in cross-sectional and cohort studies (RR: 0.67, 95% CI: 0.55–0.82). Jin et al. [28] also reported that vegetarian diet was inversely associated with prevalence of depression (odds ratio (OR): 0.57, 95% CI: 0.35–0.92, *p* = 0.023) in a study of South Asians (people from the Indian subcontinent). Vegetarian diets and their health effects are different among diverse ethnic groups and across various geographic areas. Our study provided additional knowledge in nutritional psychiatry that long-term vegetarian diets are associated with lower incidents of depression in the South-Eastern Asian population.

There are three etiological hypotheses for depressive disorders: vascular, inflammatory, and degenerative [29], and vegetarian diet appeared to play potentially important roles in all three of them:

### 4.1. Vascular

Most markers of cerebral small vessel disease (CSVD) such as white matter hyperintensity volume, subcortical infarcts, cerebral microbleeds, etc., are associated with developing new depressive symptoms in the elderly population [30]. Moreover, in a meta-analysis [31], the authors concluded that CSVD and higher levels of endothelial plasma markers, which indicate dysfunction, were associated with depression.

Chen et al. [32] demonstrated that Taiwanese vegetarians have significantly better cardiovascular risk profiles (low total serum cholesterol, low serum low-density lipoprotein (LDL) cholesterol, and low high-sensitivity C-reactive protein (hs-CRP)) than omnivores. Similar findings were found in the Adventist Study-2 [33], Black vegetarians have a significantly lower prevalence rate of major cardiovascular risk factors such as hypertension, diabetes, total serum cholesterol, LDL cholesterol, body mass index (BMI) and waist circumference, etc. when compared with non-vegetarians, and should translate into a reduced rate of cardiovascular and renal diseases.

In addition, ovo-lacto vegetarians are known to have better endothelial functions than omnivores [34], and vascular endothelial functions include regulation of vascular tone, the formation of nitric oxide (NO) and prostacyclins, the proliferation of smooth muscle cells (SMC), coagulation, fibrinolysis, the permeability of lipoproteins and plasma proteins, and adhesion and migration of blood cells and is intimately involved in early stages of atherosclerosis [35]. Thus, vegetarian diets appear to confer a protective effect on cerebral vascular disease and, hence, depression.

### 4.2. Inflammatory

Dowlati et al. [36] report significantly higher concentrations of the proinflammatory cytokines TNF-α and IL-6 in depressed subjects compared with control subjects. Moreover, high blood levels of proinflammatory cytokines, especially interleukin (IL)-8, IL-6, and tumor necrosis factor (TNF), are indeed associated with the future development of clinically significant depression [29]. On the other hand, available evidence indicates that consumption of Mg, fiber, polyunsaturated fatty acids, flavonoids, and carotenoids (which are commonly found in vegetarian diets) are associated with decreased levels of inflammatory markers such as hs-CRP, interleukin-6 (IL-6), and tumor necrosis factor alpha (TNF-α) in the serum [37]. In addition, arachidonic acid (mainly from meat) can cause a cascade of neuroinflammation that has negative effects on mood [38]. Previous studies showed that the proportion of arachidonic acid in blood samples was significantly lower in vegetarians than in omnivores since meat, fish, and poultry are the main dietary sources of arachidonic acid [39,40], thus, vegetarian diets appeared to protect against inflammation and, hence, depression.

### 4.3. Degenerative

Oxidative stress is the imbalance between the production of reactive oxygen species (ROS) and the removal of ROS, the so-called antioxidant cascade. One of the hallmarks of aging is mitochondrial dysfunction which promotes age-related disorders through increased levels of ROS [37]. ROS are known to damage all cellular biomacromolecules (lipids, sugars, proteins, and polynucleotides). The central nervous system is especially vulnerable to oxidative insult due to its low concentrations of antioxidants, high rate of O2 utilization, and high content of polyunsaturated lipids which are most susceptible to oxidation [41]. Increased oxidative stress was demonstrated in depressive disorders, and markers of oxidative stress were generally lower in vegetarians [42,43]. The antioxidant capacity involves internal factors such as glutathione and enzymes (e.g., catalase and superoxide dismutase) and external factors such as vitamins A, C, and E and phytochemicals [44]. Plant food is rich in antioxidant phytochemicals, such as phenolic acids, flavonoids, and carotenoids, and the antioxidant vitamins C and E. Indeed, vitamin C and carotenoids concentrations in blood are often reported to be higher in vegetarians compared to omnivores [45,46]. Hence, the vegetarian diet is associated with lower oxidative stress compared to meat-based diets [47], potentially conveying mood protection via this mechanism.

In addition, there are other possible mechanisms for the protective effect of a vegetarian diet on depression [48].

### 4.4. Neurotransmitters

The mood is regulated by neurotransmitters serotonin and norepinephrine. Tryptophan is the sole substrate for serotonin production. Food items that are commonly seen in the ovo-lacto vegetarian diets such as legumes, cheese, nuts, seeds, eggs, etc. are associated with higher circulating concentrations of tryptophan in the blood [49]. In addition, high circulating levels of the large neutral amino acids (LNAA), compete for entry into the brain as they use the same transporter, thus, a protein-rich meat diet causes an increase in the number of amino acids in the blood (low tryptophan–LNAA ratio) and a greater competition for the entry of tryptophan into the brain, resulting in low serotonin production. Dietary carbohydrates increase the circulating concentrations of insulin, which promotes the uptake of the large neutral amino acids into muscle cells in the postprandial period [50]. Therefore, vegetarian diets, which tend to have a high carbohydrate-to-protein ratio, may facilitate tryptophan entry into the brain and higher production of serotonin and less depression [49].

### 4.5. Gut Microbiota

The microbiota–gut–brain axis is a bi-directional communication pathway between the gut and central nervous system. It is believed to mediate or modulate various central processes through the vagus nerve and is involved with the production of microbial metabolites and immune mediators which trigger changes in neurotransmission, neuroinflammation, and behavior [51]. Studies demonstrated that there are marked alterations of the gut microbiota composition in people with depression compared to healthy controls; however, specific alterations in diversity, richness, and composition of microbiota are still not consistent [52]. The taxonomic changes observed in patients with depression are associated with the bacterial proinflammatory activity, reduced short chain fatty acids (SCFAs) production, impaired intestinal barrier integrity and neurotransmitter production, impaired carbohydrates, tryptophan, and glutamate metabolic pathways [53].

In a clinical study, probiotic administration (Lactobacillus acidophilus, Lactobacillus casei, and Bifidobacterium bifidum) in patients with major depressive disorders for eight weeks had beneficial effects on Beck Depression Inventory [54]. Short-term consumption of animal-based diet increased the abundance of bile-tolerant microorganisms and decreased microorganisms that metabolize dietary plant polysaccharides in addition to long term effect on gut microbiota enterotypes [55,56,57]. In addition, plant-based diets promote the development of more diverse and stable microbial systems which are beneficial for human health [55]. Whether plant-based diets can benefit depression risk through their effects on the microbiota–gut–brain axis and the exact mechanisms requires further study.

In addition to the diet (vegetarian or non-vegetarian), this study also revealed other risk factors for developing depressive disorders such as sex (female), lower educational attainment, and comorbidities (hypertension, diabetes mellitus, and hyperlipidemia). These results were consistent with a previous survey [58]. The risk factors involved in depression have involved a wide range of biological factors (genetic and hormonal, endocrinological) that may play a role in the underlying vulnerability (e.g., sex and physical illness) accompanied by acute life events that trigger depression [59]. Depression was also common among people experiencing chronically stressful conditions, such as socially disadvantaged and distressful relationships or lack of supportive and intimate relationships [60]. Unraveling the complex and interdependent factors of depression requires a more integrative and long-term study than what has been done or supported so far.

This study has many strengths. First, this is the first prospective study of the long-term effect of vegetarian diets on the development of depressive disorders. Second, the study consisted of a high proportion of vegetarians (~33%) with a large sample size (~10,000), which allowed us to examine the association between a vegetarian diet and the risk of depression with adequate power. Third, we assessed participants’ underlying diseases and incidental depression through the NHIRD of Taiwan which covers the entire population. As a result, the possibility of loss to follow-up has been minimized and the validity of the results has been increased. However, there were some limitations. First, the determination of the vegetarian state was self-reported from the questionnaire, so the protective effect may be underestimated in this study. Second, the definition of depression was based on the NHIRD medical claims records, and only those seeking medical help are included; subclinical depressive conditions are not explored. Third, we cannot rule out selection bias (confounding by indication) since those choosing vegetarian dietary patterns might be less likely to develop depression to begin with. However, the dietary classification and diagnosis of depression in both study groups are similar, thus does not bias the risk ratio estimates. Finally, this cohort was conducted among Taiwanese Buddhist volunteers who do not smoke nor drink alcohol; the findings, therefore, may not be generalizable to the whole population.

## Figures and Tables

**Table 1:** Socio-demographic characteristics and physical comorbidities of vegetarians and non-vegetarians.

Variables (n, %)	Non-Vegetarians	Vegetarians	*p*-Value
	(*n* = 7006, 66.2%)	(*n* = 3571, 33.8%)	
Sociodemographic characteristics			
Age, mean ± SD years	50.4 (9.7)	51.5 (9.5)	<0.01
Sex			<0.01
Male	2740 (39.1%)	928 (26.0%)	
Female	4266 (61.9%)	2643 (74.0%)	
Education level			<0.01
≤Elementary school	1455 (20.8%)	908 (25.4%)	
Middle and high school	3786 (54.0%)	1801 (50.5%)	
Higher education	1765 (25.2%)	862 (24.1%)	
Marital status ^a^			<0.01
Married	6089 (90.3%)	3037 (88.0%)	
Single	387 (5.7%)	242 (7.0%)	
Divorce or widowed	271 (4.0%)	173 (5.0%)	
Life style characteristics			
Regular exercise habit ^a^			<0.01
Regular	2366 (35.3%)	1070 (31.3%)	
Irregular	4343 (64.7%)	2354 (68.7%)	
Smoking ^a^			<0.01
Smoking	1135 (16.9%)	367 (10.7%)	
Non-smoking	5570 (83.1%)	3066 (89.3%)	
Alcohol drinking ^a^			<0.01
Drinking	1065 (16.0%)	386 (11.3%)	
Non-drinking	5573 (84.0%)	3018 (88.7%)	
Physical Comorbidities			
Hypertension	2801 (40.0%)	1265 (35.4%)	<0.01
Diabetes mellitus	1536 (21.9%)	622 (17.4%)	<0.01
Hyperlipidemia	2717 (38.8%)	1117 (31.3%)	<0.01

^a^ Some are missing data.

**Table 2:** Cox proportional hazard model for the risk of developing depressive disorders.

	Depression No.	Person-Years	IR	Model 1	Model 2 ^a^
				Crude HR (95%CI)	Adjusted HR (95%CI)
Vegetarian					
Vegetarian	78	32,898.3	2.37	0.74 (0.57, 0.96)	0.70 (0.52, 0.93)
Non-vegetarian	206	64,234.0	3.21	1.00 (Reference)	1.00 (Reference)
Age					
<50	127	46,983.0	2.70	0.86 (0.68, 1.09)	1.20 (0.90, 1.61)
≥50	157	50,149.3	3.13	1.00 (Reference)	1.00 (Reference)
Sex					
Male	68	63,487.8	1.07	0.59 (0.45, 0.78)	0.58 (0.41, 0.83)
Female	216	33,644.5	6.42	1.00 (Reference)	1.00 (Reference)
Education level					
≤Elementary school	84	21,489.6	3.91	1.94 (1.36, 2.76)	1.59 (1.07, 2.38)
Secondary school	151	51,362.1	2.94	1.46 (1.06, 2.01)	1.28 (0.91, 1.81)
College or higher	49	24,280.6	2.02	1.00 (Reference)	1.00 (Reference)
Marital status					
Single	11	5835.2	1.89	0.66 (0.36, 1.20)	0.80 (0.42, 1.53)
Divorce or widowed	19	4010.7	4.74	1.65 (1.03, 2.63)	1.54 (0.95, 2.50)
Married	241	83,814.9	2.88	1.00 (Reference)	1.00 (Reference)
Regular exercise					
Irregular	181	61,589.6	2.94	1.00 (0.78, 1.29)	1.03 (0.78, 1.35)
Regular	92	31,475.0	2.92	1.00 (Reference)	1.00 (Reference)
Smoking					
Smoking	33	13,731.4	2.40	0.81 (0.56, 1.17)	1.13 (0.69, 1.85)
Non-Smoking	235	79,400.1	2.96	1.00 (Reference)	1.00 (Reference)
Alcohol drinking					
Drinking	31	13,287.2	2.33	0.78 (0.54, 1.14)	0.98 (0.61, 1.59)
Non-drinking	236	78,943.2	2.99	1.00 (Reference)	1.00 (Reference)
Comorbidities					
Hypertension	143	37,070.5	3.86	1.64 (1.30, 2.08)	1.40 (1.05, 1.86)
Diabetes mellitus	92	19,544.3	4.71	1.90 (1.49, 2.44)	1.49 (1.10, 2.00)
Hyperlipidemia	140	32,898.3	4.26	1.71 (1.36, 2.16)	1.34 (1.00, 1.78)

Abbreviations: IR, incidence rate; HR, hazard ratio; CI, confidence interval. ^a^ All variables were adjusted for sociodemographic characteristics, life style characteristics, and physical comorbidities.

**Table 3:** The hazard ratios of depressive disorder for vegetarians compared with non-vegetarians across the different demographic groups.

	Non-Vegetarians		Vegetarians		Adjusted HR (95%CI)	*P*_interaction_
	Case No.	Person-Years	Case No.	Person-Years		
Age of baseline, years						0.97
<50	96	31,912.6	31	15,070.4	0.58 (0.37, 0.90)	
≥50	110	32,321.4	47	17,827.9	0.82 (0.56, 1.19)	
Gender						0.94
Male	55	39,118.0	13	24,369.8	0.72 (0.37, 1.39)	
Female	151	25,116.0	65	8528.5	0.73 (0.53, 1.01)	
Education level						0.15
≤Elementary school	61	13,162.9	23	8326.7	0.66 (0.39, 1.11)	
Secondary school	101	34,814.8	50	16,547.2	1.02 (0.70, 1.48)	
College or higher	44	16,256.3	5	8024.4	0.15 (0.05, 0.49)	
Marital status						0.57
Married	173	55,838.5	68	27,976.4	0.72 (0.53, 0.97)	
Single	8	3595.3	3	2239.9	0.42 (0.08, 2.12)	
Divorce or widowed	13	2432.5	6	1578.2	0.86 (0.31, 2.37)	
Regular exercise						0.44
Regular	72	21,631.2	20	9843.8	0.62 (0.37, 1.06)	
Irregular	128	39,880.1	53	21,709.5	0.77 (0.54, 1.08)	
Smoking						0.77
Non-Smoking	169	51,132.5	66	28,267.6	0.73 (0.54, 0.99)	
Smoking	28	10,355.7	5	3375.7	0.68 (0.26, 1.80)	
Alcohol drinking						0.14
Non-drinking	176	51,108.9	60	27,834.3	0.68 (0.50, 0.92)	
Drinking	21	9745.4	10	3541.8	1.12 (0.49, 2.53)	
Hypertension						0.90
No	100	38,783.4	41	21,278.3	0.70 (0.47, 1.05)	
Yes	106	25,450.6	37	11,619.9	0.73 (0.48, 1.09)	
Diabetes mellitus						0.44
No	133	50,352.0	59	27,235.9	0.77 (0.55, 1.08)	
Yes	73	13,882.0	19	5662.4	0.59 (0.34, 1.02)	
Hyperlipidemia						0.76
No	100	39,335.4	44	24,898.6	0.75 (0.51, 1.11)	
Yes	106	22,626.8	34	10,271.5	0.67 (0.44, 1.03)	

Abbreviations: HR, hazard ratio; CI, confidence interval. All variables were adjusted for sociodemographic characteristics, life style characteristics, and physical comorbidities.

## Data Availability

The data presented in the manuscript cannot be made available because it were linked to the National Health Institute Research Database (NHIRD) of Taiwan and the National Death Registry at the Health and Welfare Data Science Center (HWDSC), Ministry of Health of Taiwan. As a government regulation to protect the privacy of the participants’ personal data, all analyses were performed in the HWDSC, and only summarized results could be released.
